# Keratitis in a Dog

**Published:** 1883-07

**Authors:** 


### XI.--KERATITIS IN A DOG.

---

REPORTED FROM PROF. MOORE'S CLINIC AT THE COLLEGE.

---

A little black dog was brought in who had received an injury while playing with a kitten. The above diagnosis was made, and the disease considered of a mild type and one in which a favorable prognosis might be given.

The following treatment:—Nourishing diet, as the animal was emaciated, and the use of grs. ii. soultion of atropiæ sulphatis. The eye to be bathed with warm or cold water, which ever seemed to give the animal most relief.

The dog was also suffering from alopecia or mange, and the professor suggested that sulphuric acid be applied with a camel's hair-brush. Ultimate recovery was the final result.

